# Supplementary figures and images for: Remote monitoring of clubfoot treatment with digital photographs in low resource settings: Is it accurate?
Source: PLoS One. 2020 May 15;15(5):e0232878. doi: 10.1371/journal.pone.0232878 (PMC7228114; doi:10.1371/journal.pone.0232878)

**S1 Fig. Example of measuring clubfoot deformity from photographs.**

**
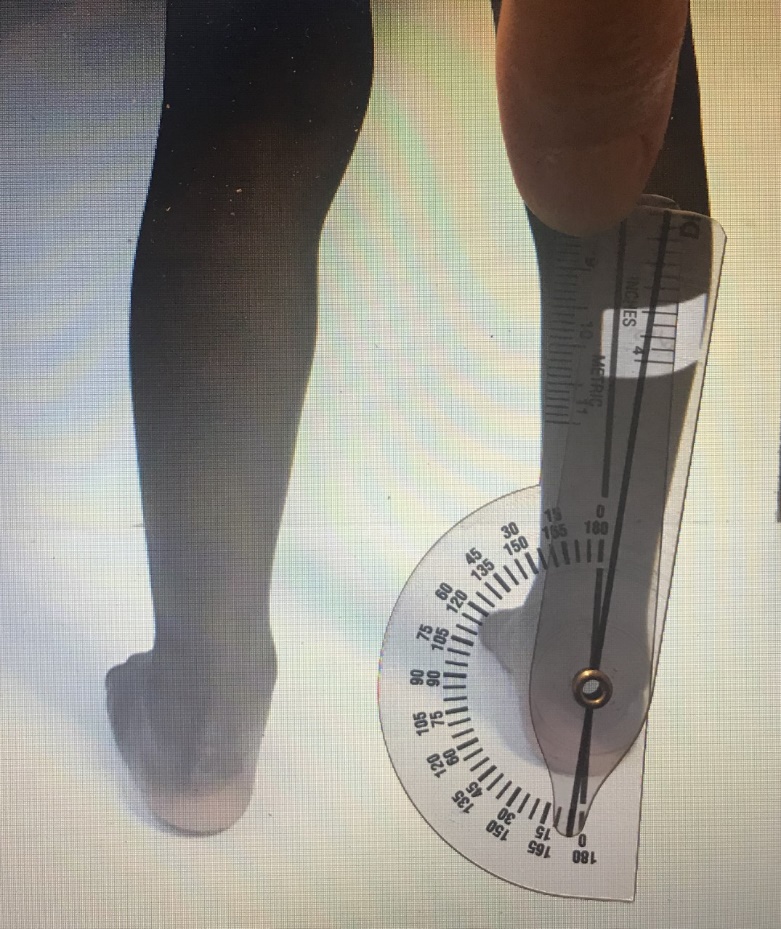
**

**
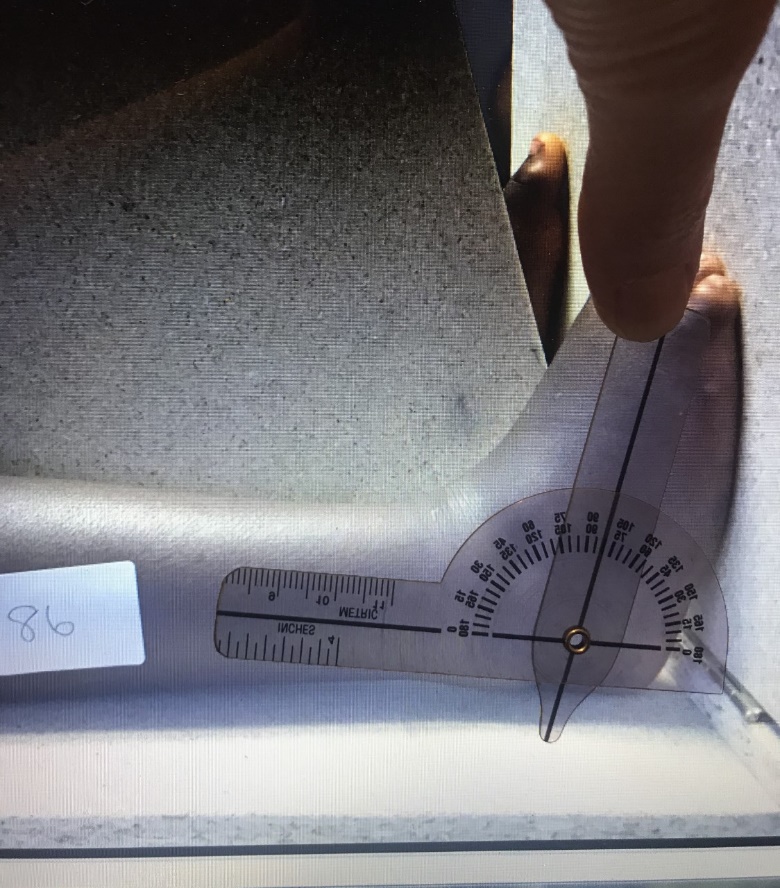
**

**
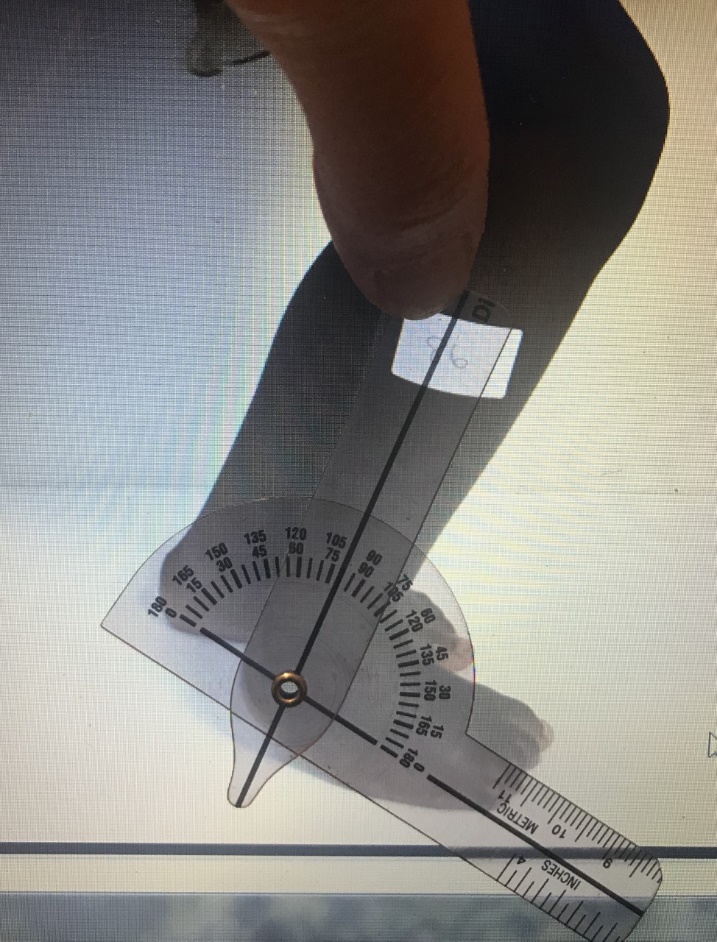
**

Supplement: S1 Fig — (DOCX) [file pone.0232878.s001.docx]
